# Laparoscopic Appendectomy for Early Appendicular Lump in Children: A Single-Institution Experience

**DOI:** 10.7759/cureus.103634

**Published:** 2026-02-15

**Authors:** Waseem Jan Shah, Mir Fahiem ul Hassan, Nisar Ahmad Bhat, Aijaz Ahsan Baba, Gowhar N Mufti, Raashid Hamid

**Affiliations:** 1 Pediatric Surgery, Sher-i-Kashmir Institue of Medical Sciences, Srinagar, IND

**Keywords:** acute abdomen in children, acute appendicitis, appendiceal mass, appendicular lump, emergency surgery, laparoscopic appendectomy, laparoscopic surgery, minimal access surgery, pediatric appendicitis, phlegmon

## Abstract

Background: The ideal management of appendicular lump in children is still debated, with traditional interval appendectomy being challenged by early laparoscopic intervention. The purpose of this study was to evaluate the safety and efficacy of emergency laparoscopic appendectomy (LA) in children with an early appendicular lump.

Methods: A retrospective chart review of 30 patients (≤15 years) presenting with an early appendicular lump who underwent emergency LA over a five-year period was performed. Outcomes measured included conversion rate, operative time, complications, and length of hospital stay.

Results: The mean age was 9.2 ± 2.8 years. The mean duration of symptoms was 3.1 ± 0.9 days. Laparoscopic approach was successful in 28 patients (93.3%). Two (6.7%) patients had to be converted to open surgery due to dense adhesions and inability to delineate anatomy clearly. The mean operative time was 68 ± 18 minutes. Operative findings included phlegmon in 18 patients (60%), contained perforation in 10 patients (33%), and abscess (≤5 cm) in two patients (6.6 %). Minor complications were seen three patients (10%). One port-site infection and two postoperative ileus occurred; all were managed conservatively. No major complications or re-operations were recorded. The mean postoperative hospital stay was 3.2 ± 1.1 days.

Conclusion: We conclude that emergency LA for early appendicular lump in carefully selected, hemodynamically stable children is a safe and feasible procedure. It offers the benefits of minimally invasive surgery while avoiding the morbidity and psychological burden of a delayed operation.

## Introduction

Acute appendicitis is the most common abdominal surgical emergency in children [1]. In approximately 2-10% of cases, it presents as an appendicular lump or mass, which represents a localized inflammatory process containing the appendix, omentum, and adjacent bowel loops [2]. The traditional Ochsner-Sherren regimen of initial non-operative management with antibiotics and intravenous fluids, followed by an interval appendectomy six-eight weeks later, has been the mainstay of treatment [3].

This approach, while avoiding difficult surgery in an inflamed field, is not without limitations. The patient is subjected to a prolonged hospital stay, the risk of recurrent inflammation or abscess formation during the interval period, the psychological burden of a planned second hospitalization [4]. Furthermore, some studies have questioned the necessity of the routine second operation [4]. With the widespread adoption and expertise in minimally invasive surgery, the paradigm is shifting. Several recent studies have suggested that early laparoscopic appendectomy (LA) in the setting of an appendicular lump can be performed safely as a single-stage definitive procedure [5,6].

The aim of this study is to present our experience with emergency LA in children presenting with an early, well-defined appendicular lump, evaluating its feasibility, safety, and outcomes.

## Materials and methods

Study design

A retrospective chart review was conducted after getting due approval by the Institutional Ethics Committee via approval number SIMS131/IEC-SKIMS/2025/175. Children (aged ≤15 years) who underwent emergency LA for a diagnosis of early appendicular lump at our tertiary care center between January 2020 and December 2024 were included. A total of 30 patients met the inclusion criteria. An "early appendicular lump" was defined by: (1) Clinical findings of a palpable, tender mass in the right iliac fossa; (2) Duration of abdominal pain ≤ 5 days; (3) Radiological confirmation via ultrasonography (USG) demonstrating an inflammatory phlegmon or a well-defined mass with or without evidence of contained perforation. 

Inclusion and exclusion criteria

We included all patients as per the diagnostic criteria above. Patients with signs of generalized peritonitis, a large abscess (>5 cm in diameter) deemed more appropriate for initial percutaneous drainage, or patients who opted for the traditional conservative approach were excluded.

Surgical technique

All surgeries were done under general anesthesia. A standard three-port laparoscopic technique was employed (umbilical 5 mm camera port, left lower quadrant 5mm working port, and right upper quadrant 5 mm port) in all patients. After diagnostic laparoscopy, the appendicular mass was carefully dissected using a combination of blunt dissection, gentle traction, and sharp division of adhesions. The mesoappendix was controlled using a vessel-sealing device. The appendiceal base was secured with Endo loops. Thorough irrigation with warm saline was performed. The appendix was extracted through the umbilical or right upper quadrant port. A tube drain was placed in the right iliac fossa in cases with significant inflammatory exudate or oozing. All patients received postoperative intravenous antibiotics (typically a second or third-generation cephalosporin with metronidazole) based on established protocols for complicated appendicitis [7], until they were afebrile with a normalizing leukocyte count, followed by oral antibiotics to complete a seven-day course. Diet was advanced as tolerated.

Data collection and statistical analysis

Data was retrieved on patient demographics, clinical presentation, laboratory and radiological findings. Operative details recorded included operative time, conversion to open and intra-operative findings. Data on postoperative complications, length of hospital stay, and follow-up were noted. Statistical analysis was performed using SPSS version 29. Variables were summarized using the mean ± SD, frequencies, and percentages.

## Results

Demographic profile and outcomes are depicted in Table 1. The study population included 30 children. These included 18 males showing a male preponderance. The mean age was 9.2 ± 2.8 years (range: 4-15 years). The mean duration of symptoms prior to presentation was 3.1 ± 0.9 days. All patients had a palpable mass on examination. USG abdomen revealed a complex mass/phlegmon in all patients. The mean operative time was 68 ± 18 minutes. Operative findings revealed a phlegmon in 18 patients (60%), a contained perforation with localized pus in 10 patients (33.3%), and a small abscess (<5 cm) in 2 patients (6.7%). Intraoperative findings, operative events, and post-operative complications are described in Table 2. In the study population LA was successfully completed in 28 patients (93.3%). Two patients (6.7%) had to be converted to open surgery due to extremely dense adhesions and friable tissues. This prevented a safe laparoscopic dissection and identification of vital structures. A tube drain was placed in four patients (13.3%) in view of diffuse oozing or substantial exudate. The drain was removed once there was no significant drain output. The average postoperative hospital stay was 3.2 ± 1.1 days. Complications were seen in three patients (10%).

**Table 1:** Demographic profile and outcomes (n=30)

Characteristic	Value
Mean Age (years)	9.2 ± 2.8
Male Patients, n (%)	18 (60)
Female Patients, n (%)	12 (40)
Mean Symptom Duration (days)	3.1 ± 0.9
Mean Operative Time (mins)	68 ± 18
Conversion to Open, n (%)	2 (6.7)
Complications, n (%)	3 (10%)
Average Hospital Stay (days)	3.2 ± 1.1

**Table 2:** . Intraoperative findings, operative difficulties, and postoperative complications in the study population (n=30)

Parameter		n (%)
Intraoperative findings	Phlegmon	18 (60)
	Contained perforation with localized pus	10 (33.3)
	Abscess <5cms	2 (6.7)
Intraoperative complications	Difficulty in locating the appendix	3 (10)
	Serosal tears to gut	2 (6.7)
	Accidental enterotomy	0 (0)
	Need for open surgery	2 (6.7)
Post-operative complications	Port-site infection	2 (6.7)
	Postoperative ileus	1 (3.3)
	Fistula	0 (0)
	Recurrent abscess	0 (0)
	Re-operation	0 (0)

These were minor complications. These included one case of port-site infection treated with wound care and oral antibiotics, and two cases of postoperative ileus that resolved with continued nasogastric decompression and supportive care. There were no major complications such as intra-abdominal abscess, fistula, or re-operation. All patients had an uneventful recovery at one month follow-up.

## Discussion

Our study adds to the growing body of literature supporting the role of early LA in selected children with an appendicular lump [5,6]. The high success rate (93.3%) and low complication rate (10% minor) in our series demonstrate that this approach is technically feasible and safe in the hands of experienced pediatric laparoscopic surgeons. Our results are consistent with a meta-analysis comparing laparoscopic and open approaches in children [8].

The mean duration of symptoms prior to presentation in our study was 3.1 ± 0.9 days. Literature reports that in patients with appendicular mass, perforated appendix, or appendicular abscess, the average duration of presentation is between three and four days [9-12]. 

The primary advantage of early appendectomy is elimination of the diseased appendix during the index admission. This avoids the potential failures of conservative management, such as abscess formation or recurrent symptoms requiring emergency intervention during the waiting period for interval surgery [4]. It also reduces the anxiety and inconvenience associated with a planned second hospitalization for both the child and parents. This is consistent with a randomized trial that found initial LA for perforated appendicitis with abscess resulted in fewer hospital days and similar complication rates compared to interval appendectomy [13].

The mean operative time in our series was 68 ± 18 minutes. It is comparable to few previously published studies [14,15]. 

The 6.7% conversion rate in our study is consistent with other studies [11]. It is an acceptable rate considering the complicated inflammatory pathology. It can be a vital step in preventing disastrous complications, especially bowel injury. Bleeding disorders and technical hurdles have been cited as reasons for conversion in some studies [16-18]. Careful patient selection is necessary. The selection criteria of symptom duration ≤5 days and the absence of a large abscess or systemic sepsis are essential. In our experience, a shorter duration of symptoms correlates with less organized and vascular adhesions, making laparoscopic dissection more feasible. This principle supports the finding that early intervention can reduce the incidence of perforations and postoperative complications [19]. The average hospital stay in our series was 3.2 ± 1.1 days conforming with other studies on LA [20]. 

The study has several limitations. It is retrospective in nature with no comparison group. The sample size is relatively small. It is a single-institution study. While our results are encouraging, they represent the experience of a center with a dedicated pediatric surgical team. A prospective randomized controlled trial comparing early LA with conventional interval appendectomy is needed to provide stronger evidence.

## Conclusions

In our experience, emergency LA is a safe and effective treatment for children with an early appendicular lump, provided they are hemodynamically stable and carefully selected. It offers the advantages of minimal access surgery while eliminating the disadvantages of the traditional delayed surgery protocol. We recommend this as a viable and preferable alternative in suitable candidates.
